# The Effects of Ultrasonic Scaling and Air-Abrasive Powders on the Topography of Implant Surfaces: Scanning Electron Analysis and *In Vitro* Study

**DOI:** 10.1055/s-0044-1782190

**Published:** 2024-05-02

**Authors:** Francesco Gianfreda, Gaetano Marenzi, Eleonora Nicolai, Maurizio Muzzi, Monica Bari, Sergio Bernardini, Daniela Adamo, Alessandra Miniello, Gilberto Sammartino, Patrizio Bollero

**Affiliations:** 1 Department of Industrial Engineering, University of Rome “Tor Vergata”, Rome, Italy; 2 Department of Neuroscience, Reproductive and Odontostomatological Science, University of Naples Federico II, Naples, Italy; 3 Department of Experimental Medicine, University of Rome Tor Vergata, Rome, Italy; 4 Department of Science, University Roma Tre, Viale G. Marconi, Rome, Italy; 5 Facoltà Dipartimentale di Medicina, Università Campus Bio-Medico, Rome, Italy; 6 Department of Systems Medicine, University of Rome Tor Vergata, Rome, Italy

**Keywords:** bicarbonate air-abrasive powders, implant–abutment junction, peri-implantitis, titanium surfaces, ultrasonic scaling

## Abstract

**Objectives** This *in vitro* study aimed to investigate the impact of bicarbonate air-abrasive powders and ultrasonic scaling with stainless steel tips on the micro- and nanotopography and roughness of three different implant–abutment junction titanium surfaces.

**Materials and Methods** Three types of sterile and decontaminated titanium surfaces (RS, UTM, XA) were used for analysis. Nine disks per surface type were subjected to micro- and nanotopography analysis, scanning electron microscopy (SEM), roughness analysis, and fibroblast cultivation. Ultrasonic debridement and air polishing were performed on the surfaces. Human dermal fibroblasts were cultured on the surfaces for 5 days.

**Statistical Analysis** Data analysis adhered to ISO 25178 standards for surface texture assessment. SEM micrographs were used to reconstruct areas for extracting roughness parameters. Excel and Mex 6.0 software were utilized for quantitative and stereoscopic analysis.

**Results** The study found varying effects on surface roughness posttreatment. RS Disco samples exhibited higher surface roughness compared with UTM and XA samples, both in average and nanoscale roughness. Decontamination led to increased surface roughness for all samples, particularly RS Disco. Fibroblast growth tests revealed enhanced cell network formation on decontaminated discs, possibly due to increased nanoscale roughness or the presence of bicarbonate salts.

**Conclusion** The study underscores the complex interplay between surface topography, microbial biofilm, and treatment efficacy in peri-implant disease management. While smoother surfaces may resist biofilm accumulation, increased nanoscale roughness postdecontamination can enhance fibroblast attachment and soft tissue integration. This dichotomy highlights the need for tailored treatment protocols that consider material-specific factors, emphasizing that successful implant therapy should balance microbial control with conducive surface characteristics for long-term osseointegration and soft tissue stability.

## Introduction

In modern dentistry is of utmost importance to ensure the stability and conservation of dental implants over time. This issue is closely linked to the prevention and treatment of peri-implantitis. Peri-implantitis, in accordance with Schwartz et al, 1 is defined as a disease affecting the tissues surrounding the implant. It is characterized by an inflammation of the connective tissues and an accelerated, nonlinear loss of supporting bone. 2 The diagnosis of peri-implantitis, proposed by Berglundh et al, consists of bone loss ≥3mm from the most coronal intraosseus portion of the implant, associated with bleeding on probing. 3

There may be many factors concerning the patient that can influence the susceptibility to peri-implantitis. 4 5 Some of them can be caused by lack of patient compliance, smoking habits, and poor oral hygiene; some other factors, like uncontrolled diabetes, history of periodontitis, and use of bisphosphonates, can contribute as they alter the host's immune response. 6 7

All the implant fixtures have a surface that can be different in material, macrogeography, and microgeography. The structure of microgeography can affect the easiness of biofilm decontamination. 8 At the International Brainstorming Meeting on etiologic and risk factors of peri-implantitis in 2014 came out that peri-implantitis is not caused solely by biofilm-associated injuries. 9 In fact, the other factors can be failed bone reconstruction, incorrect implant positioning in the three dimensions of space, abutment unscrewing, implant fracture caused by overloading, infection of internal spaces of the connection, presence of cement, incorrect prosthetic finishing line position, and the presence of deep mucosal tunnel may be considered a risk factor for peri-implant disease development and progression. 10 The implant–abutment junction is a favorable environment of bacterial growth, and the biofilm removal is not easy in the implant connection area. 5 Sometimes the prosthetic connection stability is poor, and this causes the colonization of the inner portion of the junction by the bacteria. 11 For this reason, when mucositis or peri-implantitis are established, it would be advisable to completely remove the prosthetic connection and abutment to decontaminate the entire manufact. 12 13 The microtopography of the surface can also affect the adhesion of biofilm and the cytokines proinflammatory and necrotizing release. Authors suggested that microtopographically smooth surface can promote decontamination from biofilm using ultrasound and air polishing. 14 15 16 However, this method can result in not full removal of the biofilm, because in deep pockets the effectiveness of ultrasound is lower, whereas the air polishing can cause emphysema and the use of curettes cannot guarantee the cleaning of the niches and depressions of the surface microtopography. 17 18

Aim of this *in vitro* study was to investigate the how bicarbonate air-abrasive powders and ultrasonic scaling with stainless steel tips influence the micro-, nanotopography, and roughness of three different implant–abutment junction titanium surfaces.

## Materials and Methods

### Sample Analyzed

Three different types of sterile and decontaminated surfaces (Sweden and Martina, Padua, Italy) were used for analysis.

Titanium Grade 4 surfaces were: RS (machined surface), UTM (“microgrooved” Ultrathin Threaded Microsurface), and XA (“microgrooved” Thin Machined surface).

All disks had a diameter of 10 mm and a height of 3 mm. After manufacturing, all the titanium discs underwent the same standard cleaning and sterilization procedure used for commercial dental implants.

### Sample Size

In total, 9 disks per surface were analyzed:

- Three sterile disks per surface type underwent micro- and nanotopography analyses.
- Three disks per surface type were decontaminated for scanning electron microscopy (SEM) and roughness analysis.
- Three sterile and three decontaminated disks per surface were inserted into culture with human fibroblasts to observe primary biological response.

### Ultrasonic Debridement and Air Polishing

The surfaces were then debrided using an ultrasonic device (AIR-FLOW Master Piezon; EMS) with an EMS PS Ultrasonic Tip of stainless steel (EMS, Nyon, Switzerland) under maximum irrigation and 80% power for 1 minute. The Perio-Flow nozzle (AIR-FLOW Master Piezon; EMS, Nyon, Switzerland) was directed to the nine types of implant/abutment surfaces with an angle of 60 to 90 degrees. Each surface was debrided for 30 seconds with bicarbonate powder with the dimension of 40 µm (AIR-FLOW Powder Supragingival; EMS) for two times, before and after the ultrasonic debridement.

### Fibroblast Cultivation after Ultrasonic Debridement and Air Polishing

Human dermal fibroblasts were cultured in DMEM boosted with 2 mM L-glutamine, 1% v/v pen/strep, 1,000 mg/L glucose with 10% FBS without antibiotics, in culture flasks. The tissue culture flasks were maintained at a temperature of 37°C, humidified atmosphere (CO2 5%), and split at 80% of confluence by trypsin/EDTA to obtain enough cells for the test and additional microscopic analysis of the cell surface covering. After ultrasonic debridement and air polishing, the cells were placed onto the top of the discs at the density of 190 cells/mm ^2^ and were cultured for 5 days at the same temperature and atmosphere conditions. After incubation, the samples were fixed with 2.5% glutaraldehyde in phosphate-buffered saline (PBS) and stored at 4°C until further processing. Samples were washed with PBS and deionized water and then dehydrated in ethanol solutions of increased concentration (10, 30, 50, 70, 90%) and 100% ethanol. The samples were then coated with a thin layer of gold (∼30 nm). A descriptive analysis was made by an expert in cell cultures and SEM analysis (M.M. and E.N.).

### Scanning Electron Microscopy Analysis

Samples were observed using a Gemini 300 field emission SEM (Carl Zeiss AG, Jena, Germany) and have been produced micrograph by using an accelerating voltage set at 5.0 kV and detecting secondary electrons in the Interdepartmental Laboratory of Electron Microscopy of the Rome TRE University, Italy. The samples of the nine different disc types were placed directly on plates using a double-sided carbon adhesive disc and examined by SEM.

The discs were chemically fixed and dehydrated in a graded ethanol series. Samples for the biofilm assessment were air dried in a fume hood, discs with fibroblasts were critical point dried in a CPD 030 unit (BalTec, Balzers, Liechtenstein).

Dehydrated samples, before to SEM analysis, were bonded to a plate with a double-sided carbon adhesive disc and covered with a thin layer of gold (∼30 nm) using a K550 sputter coater (Emithech, Kent, UK).

Reconstructed areas of 80 × 110 μm were used to obtain the roughness parameters, according to ISO25178. A stereoscopy of images obtained by setting the angle of inclination allowed the processing of the results through specific software (Mex 6.0, Alicona Imaging, Chicago, Illinois, United States). The three-dimensional images obtained made it possible to calculate the arithmetical mean height (Sa) at a 2000× magnification.

### Data Analysis

Data analysis was carried out using the latest version of Microsoft Excel, ensuring rigorous adherence to the ISO 25178 standard for surface texture assessment. SEM micrographs were used to reconstruct areas of 80 × 110 μm, providing a foundation for the subsequent extraction of roughness parameters. Through a comprehensive statistical analysis within Excel, the arithmetical mean height (Sa) of the surface texture was calculated at a magnification of 2000 × . The use of Excel's advanced analytical tools allowed for the facilitation of a detailed comparative analysis across the nine different disc types. The three-dimensional stereoscopic images, adjusted for the angle of inclination, were analyzed using the software Mex 6.0, which provided an additional layer of insight into the textural nuances of the samples. Each disc was evaluated for its surface roughness characteristics, with Excel serving as a pivotal tool for the visualization and interpretation of the complex data sets. This approach ensured a robust quantitative analysis, effectively bridging the gap between the high-resolution imaging capabilities of the SEM and the detailed surface texture metrics required for a comprehensive evaluation of the sample topographies.

## Results

**Fig. 1:**
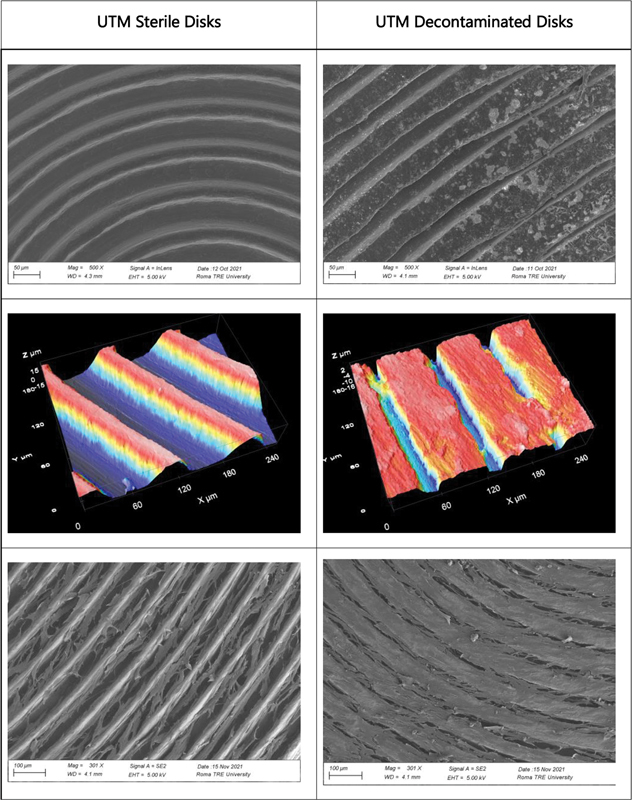
Representative images of UTM surface morphology. First row shows SEM microphotographs illustrating morphological alterations resulting from decontamination treatment. Second row shows roughness changes following decontamination treatment. Third row shows cell growth on sterile and decontaminated samples. SEM, scanning electron microscopy.

**Fig. 2:**
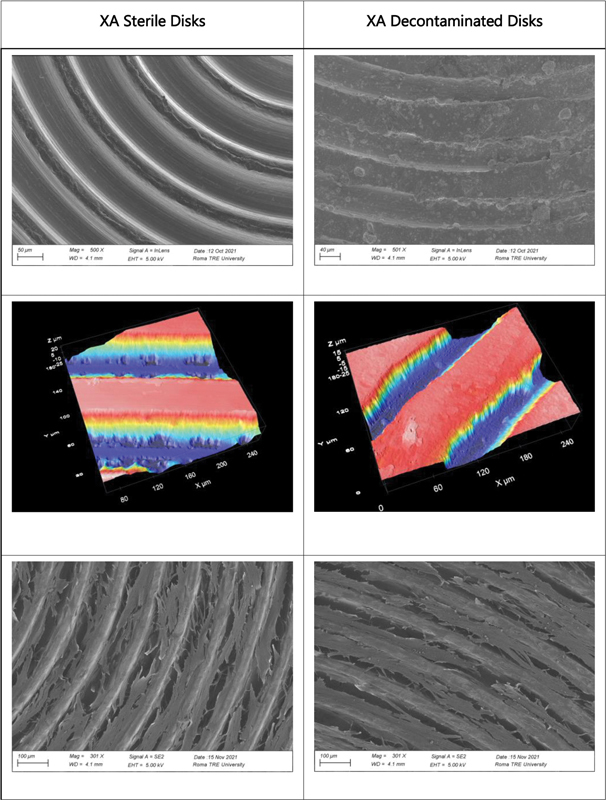
Representative images of XA surface morphology. First row shows SEM microphotographs illustrating morphological alterations resulting from decontamination treatment. Second row shows roughness changes following decontamination treatment. Third row shows cell growth on sterile and decontaminated samples. SEM, scanning electron microscopy.

**Fig. 3:**
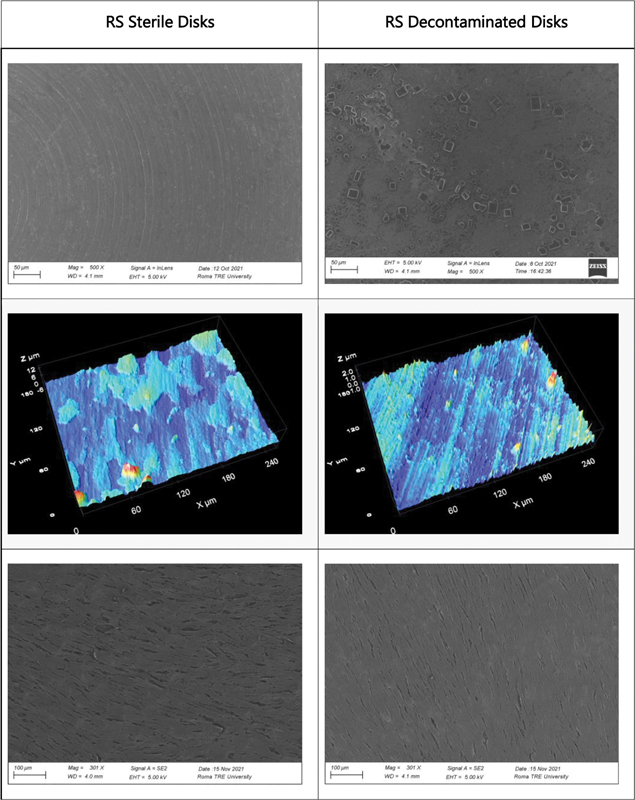
Representative images of RS surface morphology. First row shows SEM microphotographs illustrating morphological alterations resulting from decontamination treatment. Second row shows roughness changes following decontamination treatment. Third row shows cell growth on sterile and decontaminated samples. SEM, scanning electron microscopy.

The area framed by the images is about 254 × 190 µm, and the average roughness was evaluated for each sample. In particular, the UTM ( Fig. 1 ) and XA ( Fig. 2 ) samples showed a significant flattening of the peaks, which led to a decrease in average roughness. Conversely, the RS Disco samples ( Fig. 3 ), which did not initially have significant height differences, showed an increase in average roughness. Subsequently, smaller areas that only included the “upstream” or “downstream” portion of the peaks were observed to evaluate nanoscale roughness. In this case, an increase in nanoscale roughness was observed in XA, whereas the decrease in average roughness of UTM was confirmed.

Finally, for the RS Disco samples, considering a larger area, an increase in surface roughness was confirmed. Decontamination of the samples led to an increase in surface roughness for all samples but particularly for RS Disco. Specifically, decontaminated RS Disco showed an increase in surface roughness compared with the initial RS Disco. In detail, the average roughness values (Sa) ( Table 1 ) for the various samples are:

**Table 1:** Results about roughness parameters, according to ISO25178

Disc samples	Sa (254 × 190 µm area)	Sa (24 × 24 µm upstream area)	Sa (downstream area)
RS	0.252 µm	–	–
Decontaminated RS	1.108 µm	–	–
UTM	14.33 µm	883.2 nm	1057 nm
Decontaminated UTM	2.86 µm	377.2 nm	583.6 nm
XA	15.69 µm	187.2 nm	651.0 nm
Decontaminated XA	9.0 µm	320.2 nm	746 nm

Disco UTM: Sa = 14.33 µm, Decontaminated Disco UTM: Sa = 2.86 µm

Disco XA: Sa = 15.69 µm, Decontaminated Disco XA: Sa = 9.0 µm

RS Disco: Sa = 252 nm, Decontaminated RS Disco: Sa = 1108 nm

Decontaminated RS Disco: Sa = 506.12 nm

In addition, nanoscale roughness values (Sa) were evaluated for the “upstream” and “downstream” portions of the peaks of XA and UTM samples:

Disco XA “top”: Sa = 187.208 nm, Disco XA “bottom”: Sa = 651.0 nm

Decontaminated Disco UTM “top”: Sa = 320.18, Decontaminated Disco UTM “bottom”: Sa = 746.01

Disco UTM “top”: Sa = 883.208 nm, Disco UTM “bottom”: Sa = 1057.01 nm

Overall, the results indicate higher surface roughness in the RS Disco samples compared with the UTM and XA samples, both in terms of average roughness and nanoscale roughness. Furthermore, decontamination led to an increase in surface roughness for all samples, but particularly for RS Disco.

The fibroblast growth test demonstrated from a qualitative point of view in all three samples examined a greater cell network in the decontaminated discs. This could be explained by the increase in surface nano roughness or by the presence of bicarbonate salts on the surface, which could have increased the surface energy. The presence of crystal workers can in fact determine the formation of nanostructures that can mimic the texture of the extracellular matrix necessary for the adhesion of integrins and subsequently of cells.

## Discussion

The ever-increasing number of implants inserted in daily clinical practice led to an increase of related diseases as mucositis and peri-implantitis and to the need of treat them.

While peri-implant mucositis was defined as a reversible inflammatory reaction in the soft tissues surrounding a functioning implant, peri-implantitis described inflammatory reactions resulting in pathological pocket formation and loss of supporting bone around an implant in function. 19

The peri-implant mucositis is not always a reversible inflammatory reaction. The complete resolution of peri-implant mucositis after nonsurgical treatment varied from 38 to 45%. 20 21 22 23

The development of an adherent biofilm on the implant surface plays an important role in the etiology of peri-implantitis.

As a result of this multifactorial, but significant role of bacteria in the initiation and progress of infection of peri-implant diseases, elimination of the established biofilm from the implant surface is the main objective in the treatment of peri-implant mucositis and peri-implantitis. 24

These definitions may thus imply that the inflammatory process that occurs in peri-implantitis lesions is irreversible and, hence, not possible to treat. 25

At the present time, there is evidence supporting the idea that history of periodontitis smoking habits and a poor oral hygiene must be considered as risk factors for peri-implantitis. 26 27

Lately, the characteristics of biofilm in peri-implant disease have been widely examined and has been ascertained an association to a mixed anaerobic infection dominated by gram-negative bacteria and also coexisting with a high number of *peptostreptococci* and *staphylococci* . 28

According to a cause–effect view, the decontamination of implant surfaces and the disruption of bacterial biofilms was identified as essential target for the treatment of peri-implantitis. 29 To achieve this objective, several nonsurgical treatment approaches have been used, including mechanical and ultrasonic debridement, use of chemical agents (local or systemic antibiotics or local disinfectants) in addition to the previous techniques or laser application. 30 Previous controlled clinical studies pointed out that the effectiveness of nonsurgical treatment of peri-implantitis lesions was unpredictable and the clinical benefits may be limited to a period of 6 to 12 months. 31 32 33 34

These results may primarily be explained by the fact that none of the currently available methods or devices used for implant surface debridement are effective in eliminating bacterial plaque biofilms from roughened titanium implant surfaces, 32 33 35 thus impeding the establishment of a new bone-to-implant contact. 36

The application of air-abrasive devices has been suggested to get over some of these thresholds. These systems were effective to obtain complete debridement and decontamination of titanium implant surfaces, whereas their use was associated with surface alteration, which are microscopically visible. 37 38 39

These surface changings were affected by the composition of the powder, the nature and size of the particles. Specifically, powders composed with amino acid glycine (density 52.16 g/cm ^3^ ) was not associated with any alterations at moderately rough titanium implant surfaces compared with sodium bicarbonate powder (density: 51.61 g/cm ^3^ ). 39

The aim of this study was to investigate microscopically the changes, caused using bicarbonate powders and ultrasonic instruments, on the surfaces of the implant/abutment junction produced with different materials.

Many methods have been proposed in literature for decontamination of implant surfaces such as the use of plastic and metal curettes, ultrasonic instruments, air powder abrasive systems, and titanium brushes. 40

Surface microtopography always varies because of treatment but depending on the samples material an increase or decrease in roughness value has been observed. 40

In samples of UTM and XA, which have a considerable flattening of the ridges, there is a decrease of roughness, whereas in RS discs that does not present considerable gradients in the surface before the treatment, the roughness increases. However, observing smaller areas that frame only the upstream or downstream portion of the ridges, there has been an increase of nanorugosity in XA, while seems confirmed the decrease in medium roughness of UTM. For RS samples, considering the same smaller areas, with no gradients and lining on the same level, increase in roughness, observed for the larger areas, is confirmed. These data could suggest that there is not a gold standard for implant manufacturing materials and for treatment of the surface, materials with smooth surfaces can be better if we consider bacterial adhesion and cleanability, whereas the same materials could increase the roughness after treatment in case of mucositis or peri-implantitis. These results can also be compared with previous studies drawn up on fibroblasts behavior on the surfaces of the implant abutment junction to consider all the variables that can affect the long-term success of dental implants. 41

Cafiero et al 42 emphasizes the maintenance of implant surface roughness, demonstrating that all tested prophylactic procedures, especially the air powder abrasive system at a high-pressure setting (AP2), did not significantly increase surface roughness. In fact, AP2 was shown to smoothen the implant collar surface, an advantageous outcome for preventing bacterial biofilm accumulation.

In contrast, Blasi et al 43 centers on the clinical effectiveness of various instruments in removing biofilm from implant-supported restorations, concluding that nonsurgical therapy, particularly sonic scalers with plastic tips and rubber cups with polishing paste, was most efficacious in reducing peri-implant mucositis. This study, however, does not delve into the resultant physical alterations of the implant surface postcleaning. Our study presents a nuanced view where the decontamination process led to an increase in surface roughness for all samples, with RS Disco samples exhibiting a substantial increase. The observed increase in roughness, especially at the nanoscale, was associated with a qualitative enhancement in fibroblast growth on the decontaminated discs. This suggests a potential benefit of increased nanoscale roughness for cellular adhesion, which contrasts with the findings of Cafiero et al that smooth surfaces are preferable. 42 Our findings align with the clinical perspective of Blasi et al, 43 where the treatment's effectiveness is also gauged by biological outcomes, such as improved soft tissue integration indicated by enhanced fibroblast growth.

The study's scope is limited by its *in vitro* design, which may not fully capture the clinical complexities encountered *in vivo* . The surface roughness implications were examined over a short term, lacking long-term biological response data. The sample size and selection may not reflect the diversity of clinical situations, and the specific decontamination techniques evaluated do not cover the entire range used in practice. Furthermore, the positive implications of increased nanoscale roughness on fibroblast behavior necessitate further exploration to confirm their clinical significance.

## Conclusion

In conclusion, the multifaceted nature of peri-implant disease management underscores a complex interplay between surface topography, microbial biofilm, and treatment modality efficacy. While smoother surfaces, as shown by Cafiero et al, may resist biofilm accumulation, our study reveals that a certain degree of nanoscale roughness postdecontamination may be conducive to enhanced fibroblast attachment and soft tissue integration. This dichotomy highlights the necessity for a tailored approach, acknowledging that while nonsurgical therapies effectively mitigate peri-implant mucositis, as supported by Blasi et al, they may also induce variable surface roughness changes, emphasizing the need for material-specific treatment protocols. Our findings contribute to the evolving narrative that successful implant therapy must judiciously balance microbial control with the preservation of conducive surface characteristics for long-term osseointegration and soft tissue stability.
